# Food in Chronic Pain: Friend or Foe?

**DOI:** 10.3390/nu12082473

**Published:** 2020-08-17

**Authors:** Amanda C. Fifi, Kathleen F. Holton

**Affiliations:** 1 Department of Pediatric Gastroenterology, Hepatology and Nutrition, University of Miami, Miller School of Medicine, Miami, FL 33136, USA; 2 Department of Health Studies, Center for Behavioral Neuroscience, American University, Washington, DC 20016, USA

## 1. Introduction

While many still consider food to be innocuous, ongoing research demonstrates food’s role, both harmful and protective, in chronic pain. Chronic pain can stem from local organ dysfunction or can result from changes in the nervous system from central sensitization [1]. Understanding how dietary factors influence various pain conditions can help inform food-based treatments. This special issue on diet and chronic pain highlights some of the current research in this area.

## 2. Negative Effects of Diet

Glutamate, the most ubiquitous neurotransmitter in our nervous system, mediates pain transmission [2]. Thus, dietary factors which affect glutamatergic neurotransmission are of considerable interest. Free forms of the amino acids glutamate and aspartate (commonly found in flavor enhancing food additives such as monosodium glutamate (MSG) and aspartame) were associated with fibromyalgia in a case series [3]. Moreover, clinical trials restricting the consumption of additives and foods containing free forms of glutamate and aspartate resulted in significant symptom improvement in patients with fibromyalgia and irritable bowel syndrome (IBS) [4]. A study in Kenya revealed that participants with chronic pain reported improvement in pain symptoms following a low glutamate diet when compared to controls [5]. A clinical trial in Gulf War illness, which is included in this Special Issue, highlights similar results. Research also shows that MSG induces headache and masseter muscle pain when administered over 5 days [6] and the International Classification of Headache Disorders, 3rd Edition, reports that MSG is a headache trigger [7]. Furthermore, rat models revealed that visceral hyperalgesia can be reduced by blocking glutamate receptors [8]. 

Beyond the low glutamate diet, there is also growing interest in the potential role of the ketogenic diet in pain perception, due to its increasing popularity and influence on the central nervous system (CNS) for seizure control. Glucose restriction in the ketogenic diet appears to reduce neuronal excitability through restricting glucose access. This change to ketone-based metabolism may reduce nociception [9]. In animal models, maintenance on an ad libitum ketogenic diet for three weeks changed thermal nociception [10]. Diets restricting other sugars have also played a role in chronic pain relief. Avoidance of lactose is essential for preventing abdominal pain in patients with lactose intolerance [11]. Likewise, patients with chronic abdominal pain report significant symptom improvement when adhering to the low-FODMAPs (Fermentable Oligo-, Di-, Mono-saccharides And Polyols) diet with most patients (72.1%) reporting satisfaction with their symptom reduction [12].

Additionally, dietary factors which affect inflammation are also of prime importance to inflammatory pain conditions. Food additives can act directly as inflammatory mediators. Excellent examples of this include carrageenan and polysorbate 80, which are commonly added to processed foods in the US [13]. Similarly, food antigens can also cause inflammation, resulting in chronic pain. Gluten exposure in celiac patients can lead to inflammation beyond the gastrointestinal tract itself [14]. Thus, the gluten-free diet has attracted interest within the pain community [15]. Patients with rheumatoid arthritis showed improvement in their symptoms after dietary modifications which reduced immunoreactivity to food antigens [16]. In addition, patients with eosinophilic esophagitis and inflammatory bowel disease had reduced inflammation and pain relief when following specific food elimination diets [17,18].

Furthermore, the relationship between BMI and chronic pain has also been investigated. A large-scale survey of over 1 million people in the US demonstrated increased occurrence of chronic pain cases as BMI increased [19]. A review of Veterans Health Administration medical record data showed a significant association between obesity and persistent pain complaints [20]. Overweight people reported 20% greater rates of recurring pain, with rates increased to 254% for people with morbid obesity, when compared to normal weight counterparts, for pain disorders such as low back pain, fibromyalgia/chronic widespread pain, abdominal pain, and headaches [19]. Thus, food excess in general may have negative consequences for pain. Notably, being underweight also correlates with increased prevalence of chronic pain conditions. In a review of 3693 elderly patients, one in six (15.9%) experienced chronic pain, with higher prevalence in the underweight group (24.6%) than the obese group (20.2%) [21]. Thus, nutrition which supports normal body mass index (BMI) may be important for improving outcomes for patients with chronic pain. 

## 3. Positive Effects of Diet

Many ancient cultures recognized the pain-relieving properties of specific foods, and today, herbal remedies are known to have direct clinical effects on chronic pain syndromes. Animal studies found that administration of curcumin (the active ingredient in turmeric) during the early stages of peripheral neuropathy prevented the development of chronic neuropathic pain [22]. A promising study by Sharma et al. also demonstrated that curcumin mitigates thermal hyperalgesia in diabetic neuropathic pain [23], which is notoriously difficult to treat. Peppermint Mentha Piperita L. Menthacarin, the primary component of peppermint oil, blocks Ca^2+^ channels, thus causing intestinal smooth muscle relaxation to relieve chronic pain in irritable bowel syndrome [24]. In general, herbal remedies tend to elicit pain relieving properties through their antioxidant, anti-inflammatory, antiapoptotic, neuroprotective, and calcium inhibitory actions [25]. Importantly, many herbs and spices which possess these properties can easily be used daily as part of a healthy diet where foods are prepared at home.

Finally, the role of micronutrients in supporting optimal neuronal functioning is intricately linked to pain syndromes, with some nutrients having the ability to potentially modulate glutamatergic neurotransmission [26]. This Special Issue includes a review paper focusing on magnesium and chronic pain. Magnesium is thought to protect against pain based on its ability to block the NMDA (N-methyl-D-aspartate) receptor, thereby modulating glutamatergic neurotransmission [27]. Zinc is co-released with glutamate, and zinc deficiency may enhance the excitability of glutamatergic neurons [28]. Supplementation with Zn reduced inflammation and chronic pelvic pain in men with idiopathic prostatitis [29]. In a randomized control trial comparing vitamin D to a placebo, participants reported improved nonspecific musculoskeletal pain after six weeks of vitamin D administration [30]. Furthermore, in a study of 51 patients with vitamin D insufficiency and type 2 diabetes with typical neuropathic pain, vitamin D repletion (−48.5%) resulted in a significant reduction in pain scores as compared to a placebo (−39.4%) [31]. Methylcobalamin (the activated form of vitamin B12) is also being researched for its effects on pain conditions. Methylcobalamin appears to protect neurons from glutamate excitotoxicity while also facilitating regeneration of injured nerves, with analgesic effects noted in both animal and human research [32]. Thus, adequacy of micronutrient intake may also be essential for reducing pain.

## 4. Conclusions

Ongoing research continues to unveil the role that diet plays in chronic pain. Understanding how food can behave as a friend or foe will help guide future recommendations for the dietary treatment of chronic pain. We have invited an international panel of scientists, specialized in this field, to showcase such research in order to provide insight into the diet’s role in the pathogenesis and management of chronic pain syndromes.
